# Roles of extracellular vesicles in periodontal homeostasis and their therapeutic potential

**DOI:** 10.1186/s12951-022-01757-3

**Published:** 2022-12-30

**Authors:** Dao-Kun Deng, Jiu-Jiu Zhang, Dian Gan, Jie-Kang Zou, Rui-Xin Wu, Yi Tian, Yuan Yin, Xuan Li, Fa-Ming Chen, Xiao-Tao He

**Affiliations:** State Key Laboratory of Military Stomatology & National Clinical Research Center for Oral Diseases & Shaanxi Engineering Research Center for Dental Materials and Advanced Manufacture, Department of Periodontology, School of Stomatology, The Fourth Military Medical University, Xi’an, People’s Republic of China

**Keywords:** Cellular extracellular vesicles, Bacterial extracellular vesicles, Periodontal homeostasis Periodontitis, Periodontal regeneration

## Abstract

Periodontal tissue is a highly dynamic and frequently stimulated area where homeostasis is easily destroyed, leading to proinflammatory periodontal diseases. Bacteria–bacteria and cell–bacteria interactions play pivotal roles in periodontal homeostasis and disease progression. Several reviews have comprehensively summarized the roles of bacteria and stem cells in periodontal homeostasis. However, they did not describe the roles of extracellular vesicles (EVs) from bacteria and cells. As communication mediators evolutionarily conserved from bacteria to eukaryotic cells, EVs secreted by bacteria or cells can mediate interactions between bacteria and their hosts, thereby offering great promise for the maintenance of periodontal homeostasis. This review offers an overview of EV biogenesis, the effects of EVs on periodontal homeostasis, and recent advances in EV-based periodontal regenerative strategies. Specifically, we document the pathogenic roles of bacteria-derived EVs (BEVs) in periodontal dyshomeostasis, focusing on plaque biofilm formation, immune evasion, inflammatory pathway activation and tissue destruction. Moreover, we summarize recent advancements in cell-derived EVs (CEVs) in periodontal homeostasis, emphasizing the multifunctional biological effects of CEVs on periodontal tissue regeneration. Finally, we discuss future challenges and practical perspectives for the clinical translation of EV-based therapies for periodontitis.

**Graphical Abstract:**

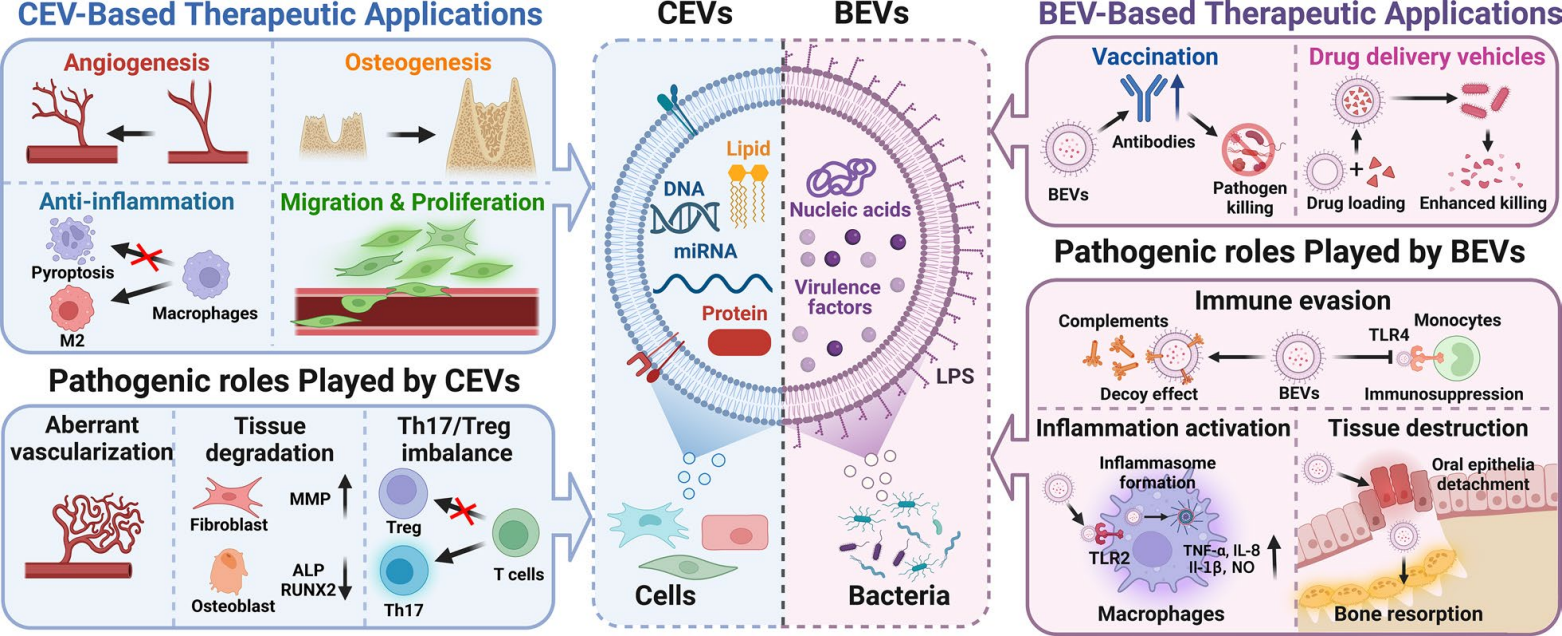

## Introduction

An estimated 50% of the world's adult population is affected by periodontitis in its mildest form, and the incidence increases to more than 60% in people over 65 years old, which makes periodontitis a disease with the sixth highest prevalence [1, 2]. Initiated and propagated by dental plaque biofilms, periodontitis can lead to continuous and irreversible destruction of periodontal tissue [3]. Left untreated, periodontitis can cause persistent alveolar bone destruction that may lead to tooth loss in adults, significantly compromising mastication, speech, and self-esteem [4]. Moreover, a significant body of evidence has indicated that periodontitis affects the overall health of patients by increasing the risk for cardiovascular disease, Alzheimer’s disease, and diabetes [2, 5]. Several strategies involving conventional anti-infection therapy (e.g., scaling or root debridement) or guided tissue regeneration have been developed for periodontal bone repair. Unfortunately, while these clinical therapies for periodontitis succeed in preventing the progression of disease, they fail to restore lost periodontal tissues stably [6].

Tooth-supporting tissues [i.e., alveolar bone, periodontal ligaments (PDLs), cementum and, gingiva] are collectively known as the periodontium. To maintain the integrity of these tissues upon microbial stimulation, the periodontium undergoes continual remodeling [7]. In a healthy periodontal milieu, typical interactions between host cells and microorganisms trigger a protective immune response, contributing to the maintenance of periodontal homeostasis. For example, commensal bacteria can activate Toll-like receptors (TLRs) on host gingival epithelial cells, leading to enhanced production of defensins or cytokines that help to maintain oral health [8]. However, in periodontal disease, enhanced interactions of host cells with pathogens can cause persistent inflammation and ultimately lead to irreversible tissue destruction. When the epithelial barrier is breached, large volumes of invading bacteria can activate TLRs on periodontal immune and nonimmune cells, leading to the robust release of many proinflammatory cytokines (e.g., PGE2, interleukin (IL)-1β, IL-8, and IL-6) and other biological mediators [8, 9]. Furthermore, the influx of bacteria and uncontrolled proinflammatory cytokines can lead to high expression of Receptor Activator for Nuclear Factor-κB Ligand (RANKL) in PDL cells and osteoblasts, causing irreversible bone destruction [10]. These findings clearly demonstrate that cell–bacteria and cell‒cell interactions are major drivers of periodontal homeostasis and play key roles in disease progression.

As communication mediators evolutionarily conserved from bacteria to humans, extracellular vehicles (EVs) produced by eukaryotic host cells (CEVs) or bacteria (BEVs) play key roles in cell–bacteria, cell‒cell, and bacteria–bacteria interactions [11, 12]. EVs constitute a group of nanosized structures composed of a lipid membrane comprising diverse proteins, nucleic acids, and lipids [12]. Once internalized by neighboring or distant cells, EVs can exert positive or negative effects, depending on their cargoes, quantity, or targeting efficiency [13–15]. Several reviews presented excellent summaries of periodontitis pathogenesis years ago [16–18]. However, they did not describe the roles of EVs in the crosstalk between bacteria and host cells, which is critical to periodontal homeostasis. Therefore, a summary of the recent advances in uncovering the pathogenesis of periodontal disease from the perspective of EVs is urgently needed.

In addition to the roles they play in cellular communication, CEVs and BEVs can be modified and used as therapeutic agents or diagnostic tools for various diseases [19, 20]. Owing to their low immunogenicity, high safety, and multifunctional bioactivities, EVs can be applied in tissue engineering fields and have therefore attracted considerable attention. An increasing number of studies have demonstrated that stem cell-derived EVs participate in immunomodulation and show the potential to serve as alternatives to stem cell-based therapies [20–22]. Notably, the therapeutic potential of EVs derived from adipose-derived stem cells in periodontitis is being investigated (ClinicalTrials.gov identifier: NCT04270006). However, the translation of EVs from the laboratory to the clinic remains challenging; for example, techniques to isolate large amounts of EVs and treatments with delayed EV clearance in vivo are needed [23, 24]. Hence, increasing tissue curative effects by combining EVs with multiphasic biomaterials is a very active area of research.

Compared with previously published reviews [22], our manuscript focuses on the roles of EVs from bacteria and cells in periodontal homeostasis, and both the therapeutic and pathogenic roles of EVs in bacteria–bacteria and cell–bacteria interactions are summarized. Our review begins with a brief overview of the biology of cellular and bacterial EVs. Then, the roles of CEVs and BEVs in periodontal homeostasis will be discussed. In this section, the pathogenic roles of BEVs in bacteria–bacteria and cell–bacteria interactions will be presented, focusing on their effects on plaque biofilm formation, immune evasion, immune activation, and tissue destruction. Afterward, the multifunctional biological effects of CEVs in periodontal homeostasis and periodontal tissue regeneration will be summarized, emphasizing the multifunctional biological effects of CEVs on periodontal tissue regeneration. Finally, the remaining challenges in EV-based therapeutic applications and some insightful perspectives on the modification and application of EVs to reconstruct the periodontium will be briefly introduced, which will benefit the development of EV-based therapies for periodontal regeneration in the future.

## Biogenesis of CEVs and BEVs

### CEV biogenesis

**Fig. 1:**
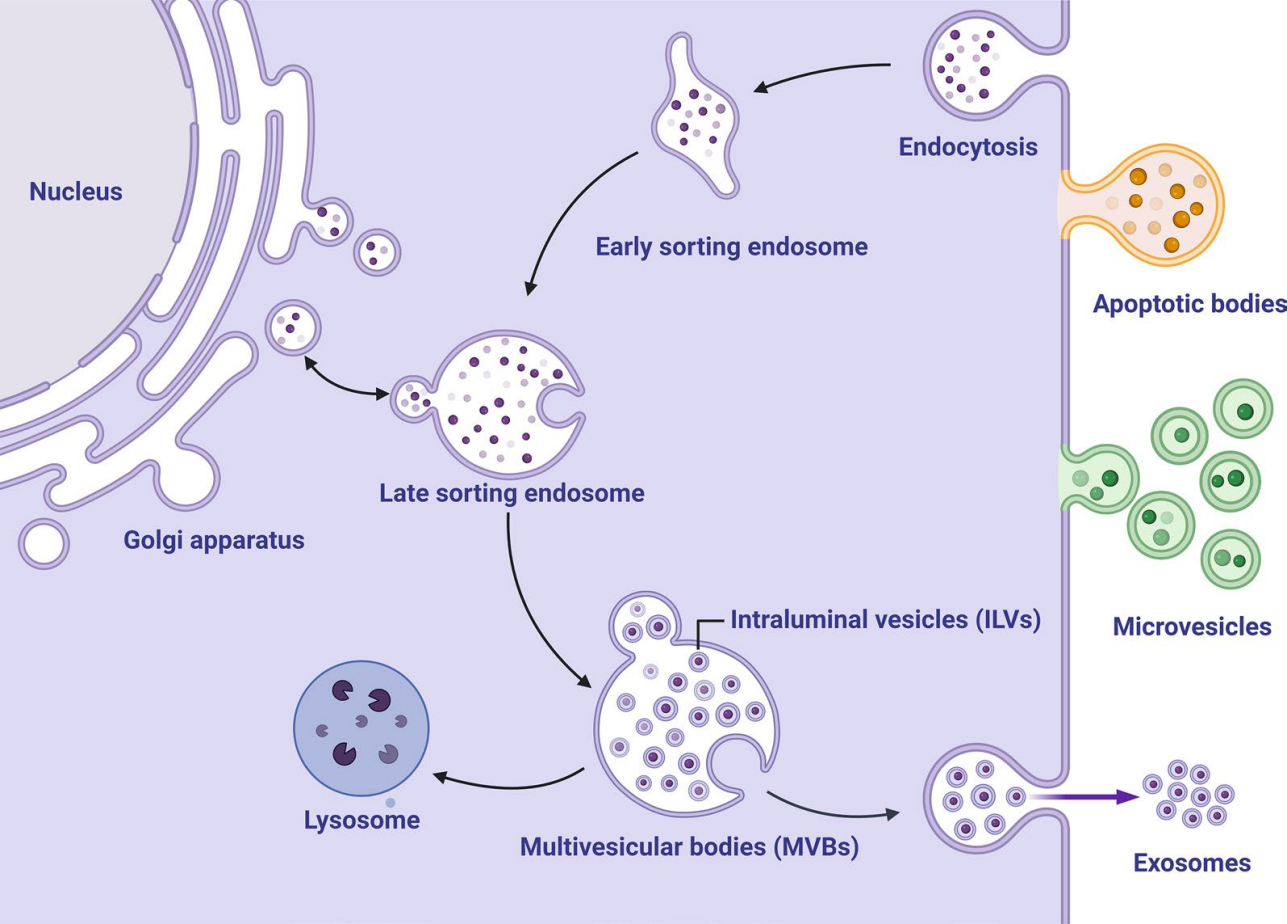
Biogenesis of extracellular vesicles (EVs) in eukaryotic host cells. Three types of extracellular vesicles have been characterized to date and are shown: apoptotic bodies (50–5000 nm) produced by apoptotic cells, microvesicles (50–1000 nm) formed through outward budding and fission of the plasma membrane, and exosomes (40–160 nm) released from eukaryotic cells via the fusion of multivesicular bodies with the plasma membrane. *ILVs* intraluminal vesicles, *MVBs* multivesicular bodies. Created with BioRender.com

Although CEVs share similar physical characteristics, they are highly heterogeneous membrane-bound vesicles that vary in biogenesis, size, and biological responses [25]. Following the nomenclature of the International Society for Extracellular Vesicles, we use the term CEVs for all membrane-bound vesicles generated by cells. According to the most up-to-date biogenesis studies on CEVs, the three main types are exosomes (40–160 nm), microvesicles (50–1,000 nm), and apoptotic bodies (50–5,000 nm). Exosomes are sequentially formed from early sorting endosomes, late sorting endosomes, and multivesicular endosomes (MVEs) containing intraluminal vesicles (ILVs). In the final step, MVEs fuse with the plasma membrane and release ILVs as exosomes [26]. Microvesicles are produced by direct outward budding and fission of the plasma membrane surface [27]. Apoptotic bodies are produced during cellular apoptosis (Fig. 1).

### BEV biogenesis

**Fig. 2:**
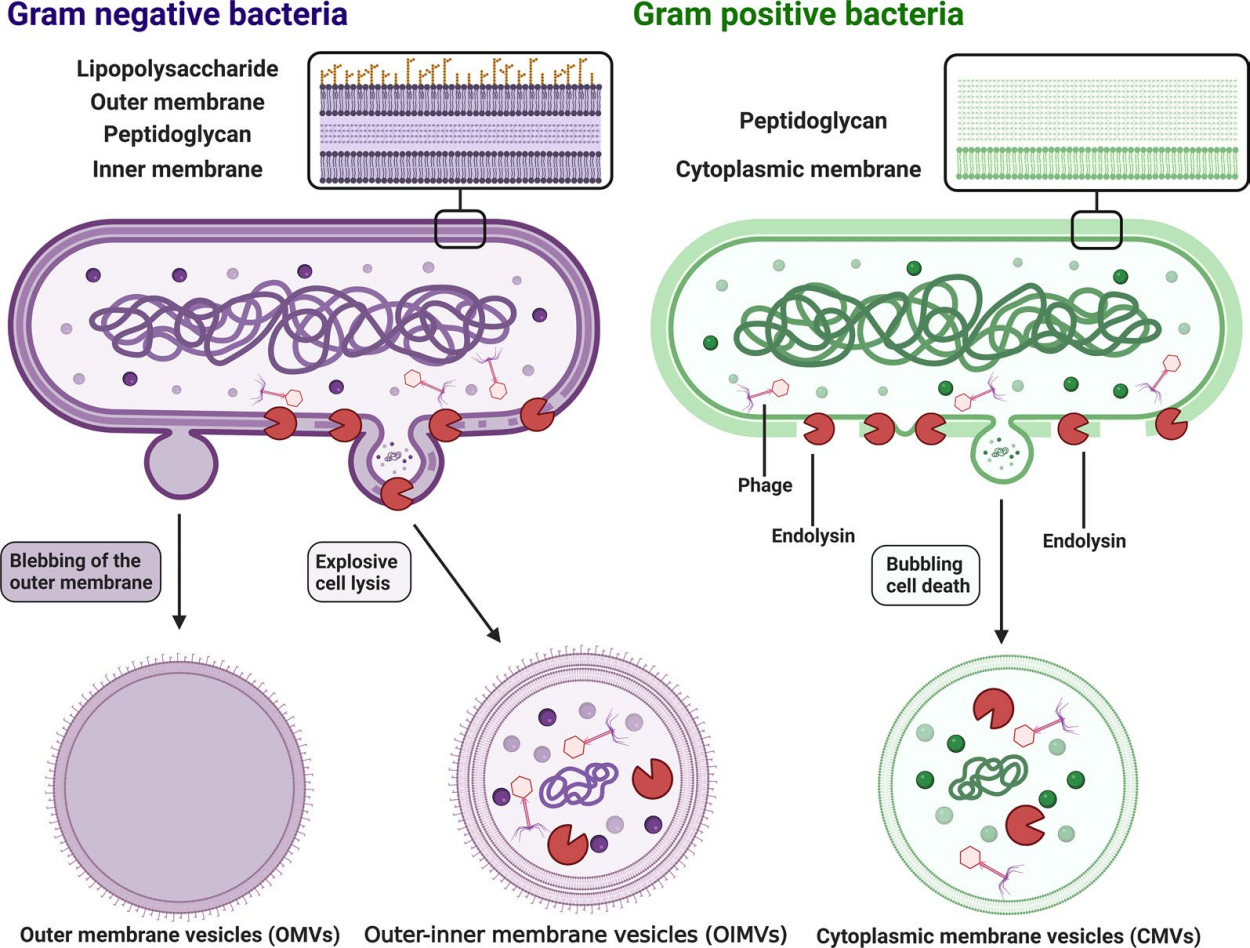
Formation of distinct bacterial membrane vesicles. Three major types of vesicles are generated by bacteria: OMVs, OIMVs, and CMVs. OMVs are produced by the blebbing of the outer membranes by living gram-negative bacteria. They do not access cytoplasmic components. OIMVs are generated by membrane fragments of dying and bursting bacteria and contain cytoplasmic components due to endolysin-induced peptidoglycan degradation. CMVs are generated by gram-positive bacteria, which lack an outer membrane. *OMVs* outer membrane vesicles, *OIMVs* outer-inner membrane vesicles, *CMVs* cytoplasmic membrane vesicles. Created with BioRender.com

Similar to eukaryotic cells, lipid membrane vesicles (20–400 nm) can also be secreted by both Gram-positive and Gram-negative bacteria. Vesicles generated by bacteria can be classically categorized into three types on the basis of their structure and composition. (1) Outer membrane vesicles (OMVs) are generated by blebbing of the outer membrane in Gram-negative bacteria. OMVs mainly comprise an asymmetrical lipid bilayer with an outer leaflet carrying many lipopolysaccharides (LPSs). Due to barriers created by peptidoglycans and the inner membrane, OMVs cannot access cytoplasmic contents and are therefore enriched with only outer membrane proteins and do not carry nucleic acids or cytosolic proteins [27, 28]. (2) Outer-inner membrane vesicles (OIMVs) are formed when autolysins or endolysins degrade the peptidoglycan layer of Gram-negative bacteria, allowing the inner membrane to protrude into the periplasmic space, which enables cytoplasmic contents to enter forming vesicles. Unlike OMVs, OIMVs contain two bilayers of membranes: one derived from the inner membrane and the other derived from the outer membrane. Among BEVs, they are the sole carriers of DNA from Gram-negative bacteria [29]. (3) Cytoplasmic membrane vesicles (CMVs) are generated by Gram-positive bacteria, which lack an outer membrane. Hence, CMVs contain cytoplasmic contents (Fig. 2) [29, 30]. In this article, the term BEVs refers to any type of vesicle released by Gram-positive or Gram-negative bacteria.

## Pathogenic roles of EVs in periodontal homeostasis

### Pathogenic roles of CEVs in periodontal homeostasis

The pathogenic roles of CEVs have been documented in a number of diseases, including Parkinson’s disease and Alzheimer’s disease [31, 32]. However, the detrimental effects of CEVs on periodontal homeostasis have not been well recognized, and most studies published thus far have concentrated on their therapeutic applications in periodontitis [33]. The maintenance of periodontal homeostasis relies on the host response to the invaded periodontopathogens [34], wherein CEVs play key roles in the development of the host response. Periodontitis shares common characteristics, including an inflammatory response, aberrant angiogenesis, and tissue destruction. Recently, emerging evidence has indicated that CEVs released by host cells can play negative roles in the host immune response and cause aberrant angiogenesis and tissue destruction. For example, CEVs released from inflamed periodontal ligament stem cells (PDLSCs) have been reported to enhance Th17-cell activation while inhibiting regulatory T-cell (Treg) activation by targeting Sirtuin-1, leading to a Th17-cell/Treg imbalance and a proinflammatory response [35–37]. Moreover, CEVs from LPS-pretreated periodontal ligament fibroblasts (PDLFs) have been found to upregulate the expression of tumor necrosis factor (TNF)-α and IL-6 in osteoblasts [38]. In addition to the host immune response, aberrant angiogenesis is a feature of periodontitis, which is closely associated with progressive inflammation. By activating miR-17-5p/VEGFA signaling pathways, CEVs from inflamed PDLSCs significantly promoted tube formation in a cell model of human umbilical vein endothelial cells (HUVECs) [39], indicating that they may contribute to anomalous vascularization in periodontitis. With the progression of inflammation, tissue destruction is inevitable, and there have been reports of EVs being involved in tissue degradation. For example, EVs released from PDLFs pretreated with LPS have been found to inhibit osteogenesis [38]. Furthermore, EVs released from biofilm-stimulated gingival epithelial cells significantly upregulated MMP-1 and MMP-3 expression in human gingival fibroblasts (HGFs). Upregulated MMP-1 and MMP-3 in HGFs promoted a tissue-destructive phenotype and reduced extracellular matrix (ECM) production, leading to increased tissue degradation [40].

In summary, CEVs play essential roles in modulating the host immune response and related tissue destruction. Despite these findings, the harmful effects of host cell-derived EVs on periodontitis-induced bone resorption are still unknown. Therefore, the pathogenic role of CEVs in periodontitis is largely unclear, and more research is required to investigate the roles of CEVs in periodontitis pathogenesis.

### Pathogenic roles of BEVs in periodontal homeostasis

Compared with CEVs, BEVs have been widely recognized as carriers of the bacterial virulence repertoire, which plays a role in the pathogenesis of various diseases [41–43]. Indeed, an increasing body of evidence has indicated that BEVs generated by periodontopathogens initiate and accelerate the progression of periodontal disease by delivering a large number of virulence factors, including LPS, lipids, and outer membrane proteins, to host cells [27]. By interacting with other bacteria and host cells, BEVs not only promote the survival and evasion of periodontopathogens, but also exacerbate inflammation and cause periodontal destruction [44].

#### Enrichment of virulence factors

Since BEVs are mainly derived from the membrane of bacteria, their structures are composed of phospholipid-rich membranes that can carry large amounts of bacteria-derived virulence factors. These virulence factors include membrane-associated molecules (e.g., LPS and gingipain), cytosolic proteins (e.g., peptidyl arginine deiminase), and nucleic acids (e.g., RNA and DNA) [45, 46]. BEVs enhanced the pathogenic effects of their parent bacterial cells by carrying highly concentrated virulence factors [47, 48]. Compared with the effect of parent *Porphyromonas gingivalis* cells, BEVs released from *P. gingivalis* showed much higher invasive activity [49] and resulted in higher expression of cytokines, including IFN-β, IL-12p70, IL-6, IL-10, and TNF-α, in macrophages. Moreover, BEVs activated inflammasome formation through caspase-11 and led to macrophage pyroptosis, but their parent bacteria could not exert similar effects [50]. The enhanced pathogenic effects of *P. gingivalis* BEVs are not entirely due to the asymmetric bilayer with LPS exposed on the outer leaflet, since they were also effective in increasing IL-1β secretion and activating the formation of inflammasomes in monocytes, which cannot be attributed to LPS alone [51]. In addition to *P. gingivalis* BEVs, compared to their parent bacterial cells, BEVs from *Tannerella forsythia* induced higher expression levels of monocyte chemoattractant protein-1 (MCP-1), IL-8 and IL-6 in PDLSCs and led to higher TNF-α and IL-8 expression in macrophages [52].

In summary, periodontopathogens can exert more devastating effects during periodontitis progression when virulence factors are enriched in BEVs. However, many important but unanswered questions about the increased pathogenic effects of BEVs remain. For example, why do BEVs generated by periodontopathogens present enriched virulence factors, and what are the mechanisms that lead to this enrichment? Further investigation is urgently needed to discover the underlying mechanisms of BEV-mediated enrichment of virulence factors. The answers may contribute to our understanding of bacteria-driven diseases, including but not limited to periodontitis.

#### Plaque biofilm formation

Although the etiology of periodontitis is still unknown, the formation of plaque biofilms is considered to play central roles in initiating and accelerating the development of periodontitis [53]. The plaque biofilm-encapsulated ECM protects bacteria from various external stimuli and countering mechanisms in host cells that disperse and eradicate bacteria. Increasing evidence has indicated that BEVs participate in plaque biofilm formation by promoting bacteria–bacteria interactions, aiding in bacterial adhesion to host epithelial cells, and promoting the transport of nonmotile bacteria [54]. Due to the BEV-packed gingipains or adhesin enrichment, *P. gingivalis* BEVs significantly increased the coaggregation of other bacteria (e.g., *Staphylococcus aureus* and other *Streptococcus* spp.) on the tooth surface and in gingival crevices [55]. In addition, *P. gingivalis* BEVs facilitated the attachment and invasion of other bacteria (e.g., *T. forsythia*) on host epithelial cells [56]. Furthermore, *P. gingivalis* BEVs enabled the coaggregation of nonmotile bacteria with motile bacteria (e.g., *Treponema denticola* and *Lachnoanaerobaculum saburreum*), which led to the movement of nonmotile bacteria with motile bacteria to form polymicrobial biofilms [57]. The aforementioned studies support the findings that BEVs from periodontopathogens contribute to bacterial aggregation and plaque biofilm formation.

#### Immune evasion

**Fig. 3:**
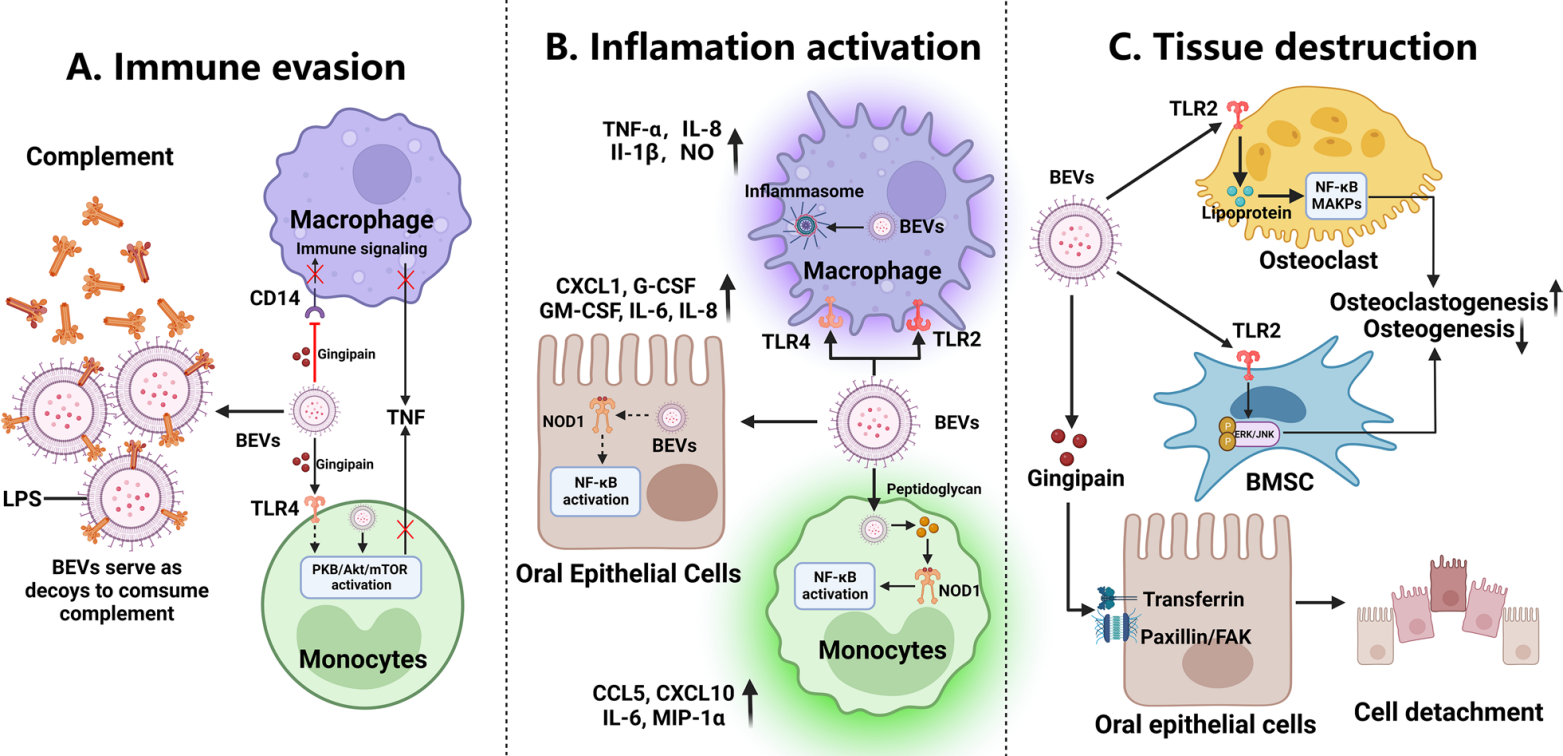
Role of extracellular vesicles (EVs) and bacterial extracellular vesicles (BEVs) in the pathogenesis of periodontitis. **A** The effects of BEVs on immune invasion. BEVs can serve as decoys that disrupt the complement system, thereby protecting periodontopathogens from complement bactericidal effects. Moreover, BEVs can suppress microbial recognition by macrophages/monocytes by interacting with host cell membrane receptors. **B** BEVs released from periodontopathogens can bind to the pathogen recognition receptors (PRRs) of host cells, including TLR2, TLR4, and NOD1, triggering the activation of downstream proinflammatory signalling pathways. **C** BEVs released from periodontopathogens cause periodontal tissue destruction. BEVs can deliver virulence factors, including gingipain and cytolethal distending toxin (CDT), to oral epithelial cells, causing cell dysfunction and detachment. Additionally, BEVs can inhibit the osteogenesis of bone marrow stromal cells (BMSCs) and induce the osteoclastogenesis of osteoclast precursors, leading to bone tissue destruction. *BEVs* bacterial extracellular vesicles, *PRR* pathogen recognition receptor, *TLR2* Toll-like receptor 2, *TLR4* Toll-like receptor 4, *NOD1* nucleotide-binding oligomerization domain-containing protein 1, *CDT* cytolethal distending toxin, *BMSCs* bone marrow stromal cells. Created with BioRender.com

BEVs facilitate bacterial survival by invading the host, triggering the host response during periodontitis in numerous ways. First, BEVs can serve as decoys, protecting invading pathogens from bactericidal factors such as complement system compounds. For example, BEVs from either *A. actinomycetemcomitans* or *P. gingivalis* suppressed the activity of bactericidal factors in human serum, and BEVs released from *A. actinomycetemcomitans*, a complement immune system target, consumed complement components in an LPS-dependent manner, thereby shielding the orgnism from the bactericidal activity of complement factors [58, 59]. Second, BEVs can inhibit the functions of immune cells, thereby facilitating bacterial evasion of host immune defense. For example, BEVs released from *P. gingivalis* degraded the LPS-detecting CD14 receptor on human macrophage-like cells, reducing proinflammatory signaling in a gingipain-dependent manner [60]. Moreover, gingipains in *P. gingivalis* BEVs recognized Toll-like Receptor 4 and activated the PI3K/AKT/mTOR pathway in monocytes to induce selective TNF deficiency, hampering immune cell recognition of microbes (Fig. 3A) [61]. Third, in addition to evading innate immune cells (e.g., macrophages and monocytes), BEVs can facilitate periodontopathogen evasion of adaptive immune cells, such as T cells. Notably, small RNAs (similar in size to miRNAs) carried by BEVs released from periodontopathogens (e.g., *A. actinomycetemcomitans* or *P. gingivalis*) suppressed the production of certain cytokines, including IL-13 and IL-5, by Jurkat T cells, suggesting that signaling molecules carried by BEVs mediate immune evasion via bacteria-to-human interactions [62]. In summary, these findings suggest that BEVs aid in periodontopathogen evasion of the host immune system by serving as decoys to reduce bactericidal factor effectiveness and inhibiting the function of immune cells, thus favoring the survival of periodontopathogens.

#### Activation of inflammatory responses

Initiated by plaque biofilm bacteria, periodontitis is characterized by persistent tissue-destroying inflammation [63–65]. Since they can be enriched with virulence factors, BEVs can activate and amplify the host immune response in a manner similar to their parent bacteria. As the first line of defense against infection, the oral mucosal epithelium can be activated by BEVs to trigger innate immune signalling in multiple ways (Fig. 3B) [66]. The surface-exposed LPS or porins/lipoproteins on the outer leaflets of OMVs bind to epithelial cell pathogen recognition receptors (PRRs), including TLRs, and activate downstream proinflammatory signaling pathways [67]. For example, BEVs released from *Filifactor alocis,* a newly discovered periodontopathogen, activated TLR2 in human oral epithelial cells and enabled at least a 1.5-fold increase in the expression of proinflammatory cytokines, including IL-6, IL-8, G-CSF, GM-CSF, and chemokine (C-X-C motif) ligand-1 (CXCL-1) [68, 69]. When BEVs enter epithelial cells, virulence factors (e.g., peptidoglycan) carried by BEVs can activate an intracellular PRR, nucleotide-binding oligomerization domain-containing protein 1 (NOD1), leading to innate immune responses in epithelial cells [70]. For example, peptidoglycan delivered into host cells by BEVs activated NF-κB responses via a NOD-dependent mechanism and resulted in increased induction of innate immune signaling [71].

When BEVs pass the oral epithelial barrier (i.e., the gingival epithelium) and enter the underlying submucosal tissues, they interact directly with host immune cells, such as dendritic cells, monocytes and macrophages [72], leading to the activation of innate or adaptive immune responses [73, 74]. Similar to those on epithelial cells, the PRRs on host immune cells, including TLRs and NOD-like receptors, recognize BEV-carried danger-associated molecular patterns (e.g., LPS, peptidoglycans, proteins and nucleic acids) to trigger a cascade of inflammatory responses [71, 75, 76]. For example, BEVs produced from the periodontopathogenic “red complex”, consisting of *P. gingivalis*, *T.* forsythia and *T. denticola,* bound to PRRs on macrophages and monocytes, thereby leading to excessive activation of NF-κB and increased TNF-α, IL-1β, and IL-8 production [77]. Moreover, the BEVs produced by different parent bacterial cells in “red complexes” stimulated PRRs to different degrees. Compared with those released from either *T. denticola* or *T. forsythia*, BEVs released from *P. gingivalis* induced stronger TLR2- and TLR4-specific responses and more moderate responses in cells expressing NOD2, NOD1, TLR8, TLR7, and TLR4, confirming the dominant roles of *P. gingivalis* and related BEVs in the pathogenesis of periodontitis. In contrast, BEVs released from *T. denticola* or *T. forsythia* induced only weak responses [77]. When entering immune cells, the BEVs released from *A. actinomycetemcomitans* promoted the induction of cytoplasmic peptidoglycan sensor activity and led to NOD1-dependent NF-κB activation in monocytes, which significantly increased the production of chemokine (C–C motif) ligand (CCL)-5, CXCL-10, IL-6 and macrophage inflammatory protein (MIP)-1α [78]. These findings strongly suggest that BEVs are important pathogenic factors of bacteria, and the roles of BEVs in the development of periodontitis may differ from each other. Moreover, the ways that BEVs activate PRPs in immune cells determine downstream immune response signaling. However, most of these findings were based on studies performed in vitro, and further investigations under in vivo conditions are still needed.

#### Destruction of periodontal tissues

In addition to their activating effects on inflammation, periodontopathogens can produce various virulence factors that are involved in periodontal tissue destruction. As mentioned in the previous section, BEVs released from periodontopathogens can serve as vehicles for high concentrations of toxins, including gingipains and cytolethal distending toxin (CDT), causing dysfunction and detachment of host oral cells [79]. For example, *P. gingivalis* BEV-associated gingipains could compromise the function of epithelial cells by degrading integrin-related molecules and transferrin receptors [80, 81]. Moreover, the BEV-encapsulated gingipains could disrupt the fibronectin-integrin interactions in HGFs, leading to the formation of periodontal pockets [82]. CDT is associated with cell cycle arrest and apoptosis. The BEV-mediated release of CDT killed HGFs, possibly damaging the sulcular/junctional epithelium [83]. Apart from negatively affecting soft tissue, BEVs can transfer toxic components to osteoblasts and osteoclasts, leading to periodontal bone dyshomeostasis. By activating the TLR2 downstream signaling of the ERK and JNK pathways, BEVs released from *F. alocis* inhibited osteogenic differentiation and increased the RANKL/OPG ratio in bone marrow stromal cells (BMSCs) to promote osteoclast differentiation, resulting in increased bone resorption [84, 85] (Fig. 3C). Furthermore, increasing evidence has indicated that BEVs lead to periodontal bone loss indirectly by inducing hyperactivation of the immune response. BEVs released by *F. nucleatum* could induce M0-like macrophage switching to M1 macrophages, significantly increasing the production of proinflammatory cytokines. Moreover, in vivo studies have indicated that *F. nucleatum* BEVs increased osteoclast numbers and aggravated alveolar bone loss in a mouse model of periodontitis [86]. Overall, BEVs can transport virulence factors to host cells, impairing oral epithelial barrier structure and accelerating periodontal bone loss, thereby resulting in periodontal tissue destruction.

#### Development of systemic disease

Due to protection conferred by vesicle membrane structures, BEVs can diffuse into the bloodstream, enter host cells, and transfer toxic components to distant organs. Therefore, BEVs secreted by periodontopathogens not only contribute to the development of periodontitis but also play relevant roles in the progression of a variety of systemic diseases, including Alzheimer’s disease, diabetes, and cardiovascular disease, which may explain to some extent the involvement of periodontitis in other systemic diseases [87, 88].

Mounting evidence has indicated that *P. gingivalis* BEVs are risk factors for cardiovascular diseases such as atherosclerosis, wherein endothelial dysfunction, endothelial permeability, and calcium deposits play key roles during atherosclerosis development. Compared with parent bacteria, nanosized OMVs could easily migrate in the blood vessels where the parent bacteria cannot reach, causing vascular damage and contributing to the atherosclerosis process [89]. *P. gingivalis* BEVs have been reported to aggravate endothelial dysfunction by activating Rho kinase-induced ERK1/2 and p38 MAPK pathways [90]. Researchers have found that *P. gingivalis* BEVs decreased the expression of the endothelial adhesion molecule PECAM-1 (CD31), leading to enhanced endothelial permeability in vitro and in vivo [91, 92]. Furthermore, BEVs secreted from *P. gingivalis* activated the ERK1/2-RUNX2 pathway to induce calcification of vascular smooth muscle cells, thereby accelerating the development of atherosclerosis [93].

Alzheimer's disease, an inflammatory neurodegenerative condition, is characterized by myeloid plaques and neurofibrillary tangles. Accumulative evidence has shown that periodontitis is closely linked to Alzheimer's disease, suggesting that periodontitis may play a role in the progression of Alzheimer's disease via the transfer of periodontopathogenic BEVs [94]. For the first time, Han et al. demonstrated that BEVs from *A. actinomycetemcomitans* crossed the blood‒brain barrier and reached the brains of mice. By delivering extracellular RNA cargoes, BEVs significantly increased the expression of TNF-α [95]. The BEV-mediated integrity loss and permeability of the human blood‒brain barrier have also been reported by other researchers [96]. Furthermore, the Han et al. group found that BEVs released from *A. actinomycetemcomitans* could be taken up by brain immune cells, such as macrophages and microglial cells. In vitro experiments have shown that the extracellular RNA cargoes carried by BEVs enhanced the expression of IL-6 in microglial cells by activating NF-κB pathways [95, 97].

The metabolic disorder diabetes mellitus is characterized by elevated blood glucose. BEVs released from *P. gingivalis* can be delivered to the liver, where they accumulate, significantly reducing the synthesis of hepatic glycogen, attenuating insulin sensitivity, and resulting in high blood glucose levels. Mechanistically, gingipains in *P. gingivalis* OMVs could negatively regulate Akt/GSK-3β pathway activation, thereby attenuating insulin sensitivity and glycogen synthesis in liver cells [98].

In summary, these studies reveal a novel mechanism through which systemic diseases and periodontitis are closely related, implying that BEVs play vital roles in the association between systemic disease and periodontitis. However, clinical evidence and more detailed mechanistic studies are still required to test and validate these findings.

## CEV-based therapeutic applications in periodontal regeneration

Periodontitis is characterized by continual and irreversible destruction of tissues supporting the tooth. Regenerating the lost periodontium, including the PDLs, alveolar bone, and cementum is the ultimate goal of periodontal treatment [3, 99]. Due to their regenerative capability and immunomodulatory effects, stem cell-based strategies are among the most promising regenerative therapies for periodontal regeneration [3, 100, 101]. However, stem cell-based therapies are limited due to great challenges, including the high cost of cell expansion, uncontrolled cell proliferation, the potential for tumorigenesis, and immunogenicity [102, 103]. Moreover, maintaining cell viability and controlling cell differentiation following cell transplantation in situ have yet to be achieved due to the complex periodontal microenvironment [104]. Mounting evidence has indicated that the therapeutic effects of stem cells are mainly derived from the release of CEVs [105, 106], and that CEVs can recapitulate the therapeutic effects of their parent cells [107, 108]. Therefore, stem cell-derived EV therapies are potential substitutes for stem cell-based therapies [109, 110]. Compared with those of their parent stem cells or other biomolecules, CEV-based regenerative therapies offer the following key advantages: (1) CEV-based strategies pose fewer risks, including unwanted transformation and immunogenicity [111]; (2) CEVs exert multifunctional effects to target diverse therapeutic mechanisms due to their cargoes, including abundant proteins, nucleic acids, and lipids (Table 1). Herein, we summarize the multifunctional biological effects of dental stem cell-derived CEVs on periodontal regeneration.

**Table 1:** CEV-based therapeutic applications in periodontal regeneration

CEV origin	CEV cargos	Involved pathway	Administration methods	Functional effects	Refs
PDLSC	miR-590-3p	miR-590-3p/TLR4	Local injection	Inhibit macrophage pyroptosis	[113]
DPSC	miR-1246	NF-κB p65, p38 MAPK	Chitosan hydrogel	Promote anti-inflammatory macrophage phenotype	[114]
TNF-α treated GMSC	CD73, miR-1260b	miR-1260b/Wnt5a/RANKL, JNK	Local injection	Promote macrophages toward M2 polarization, inhibit osteoclastogenesis	[116]
MSC	CD73	AKT, ERK	Collagen sponge	Promote PDL cell migration and proliferation	[118]
SCAP	Cdc42	–	Local injection	Promote angiogenesis	[119]
SHED	Wnt3a, BMP2	AMPK, Wnt/β-catenin, BMP/Smad	β-TCP, Local injection	Promote osteogenesis and angiogenesis, repress inflammatory cytokines expression	[110] [120] [121]
PDLSC	Wnt	–	β-TCP, Matrigel	Promote osteogenesis	[123]
LPS-Preconditioned DFC	Wnt3a, BMP2	RANKL/ OPG	Gelatin/laponite hydrogel	Promote PDLC proliferation, migration, and osteogenic differentiation, inhibit osteoclast formation	[127]

### Anti-inflammatory effects

**Fig. 4:**
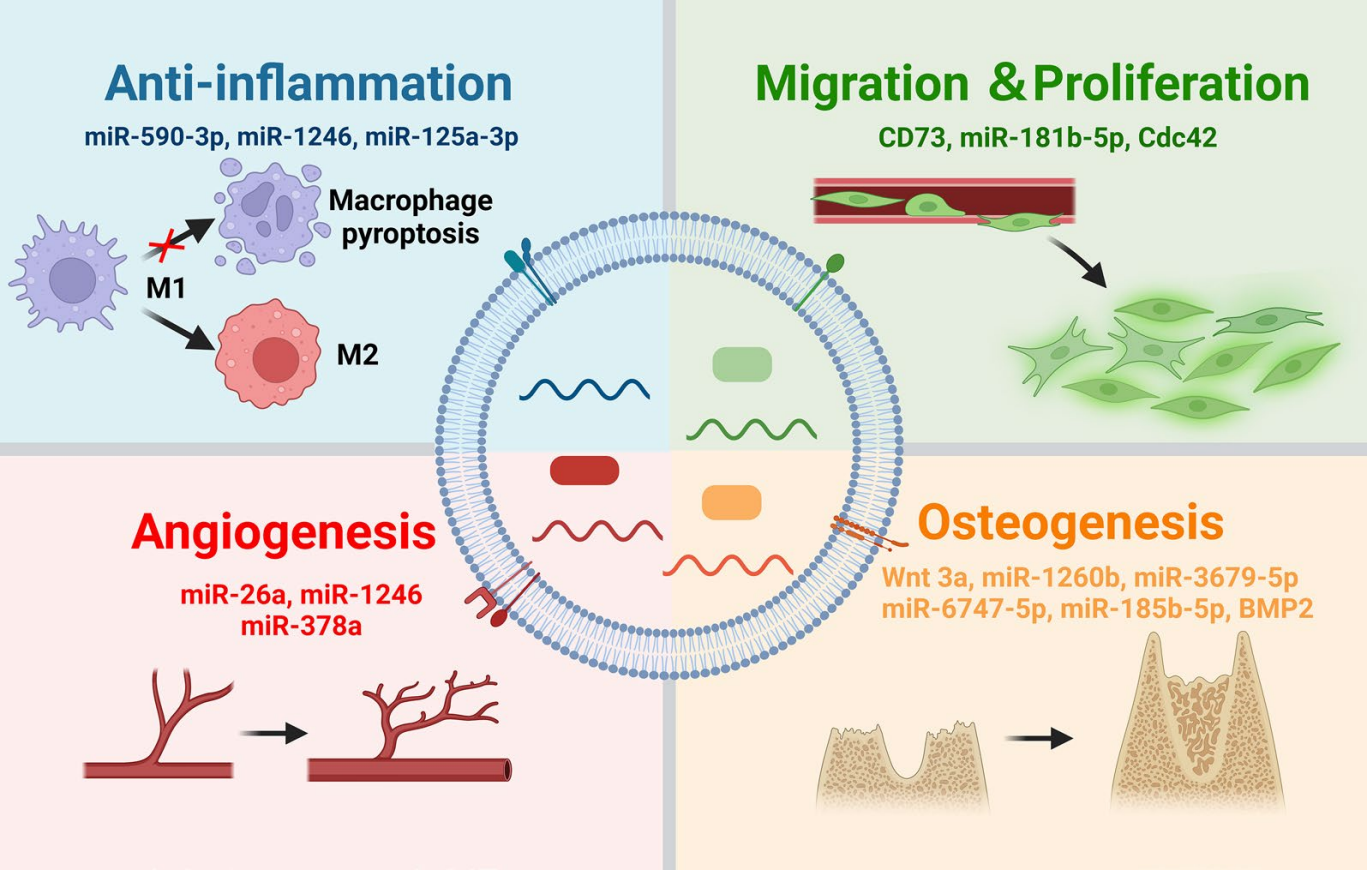
The bioactive effects of CEVs and their cargoes on anti-inflammatory responses, angiogenesis, cell migration and proliferation, and osteogenesis. Functional nucleic acid and protein cargoes carried by CEVs are classified into four panels on the basis of their effects. Created with BioRender.com

The resolution of inflammation is a prerequisite for periodontal tissue regeneration [112]. CEVs released from various types of oral stem cells have shown anti-inflammatory abilities, contributing to effective periodontitis treatment (Fig. 4). For example, CEVs released by stem cells from human exfoliated deciduous teeth (SHED-EVs) repressed IL-6 and TNF-α gene expression in bone marrow mesenchymal stem cells (BMMSCs) and prevented alveolar bone loss in mouse models of periodontitis [110]. In a similar study based on a murine model of periodontitis, CEVs released from PDLSCs decreased the production of IL-18, TNF-α, and IL-1β to alleviate bone loss in periodontitis. Mechanistically, PDLSC-derived CEVs expressed a stable level of miR-590-3p, which can suppress TLR4 downstream signaling pathways, thereby inhibiting macrophage pyroptosis [113]. In addition, CEVs released from dental pulp stem cells (DPSC-EVs) induced macrophage polarization to an anti-inflammatory phenotype. The immunomodulatory effects of DPSC-EVs were mainly the result of miR-125-3p and miR-1246 carried by DPSC-EVs. By incorporating chitosan, these DPSC-EVs accelerated the healing of the damaged periodontal epithelium and alveolar bone [114, 115].

Since CEV-encapsulated bioactive molecules, including nucleotides, proteins, or lipids manifest the biological effects of CEVs, strategies to change the content compositions of CEVs may significantly enhance the anti-inflammatory functions of CEVs. To this end, Nakao et al. reported that TNF-α pretreatment increased not only the amount of CEVs secreted from gingival tissue-derived MSCs (GMSCs) but also the immunomodulatory effects of GMSC-EVs by inducing a higher anti-inflammatory M2 macrophage polarization rate. Mechanistically, highly expressed exosomal CD73 in TNF-α-pretreated GMSC-EVs is responsible for enhanced M2 polarization [116]. In another report, cyclic stretch force enhanced the inhibitory effects of PDL-derived CEVs on IL-1β production in LPS-stimulated macrophages by suppressing the nuclear translocation of NF-κB and the binding of NF-κB-p65 to DNA [117]. These findings suggest that preconditioning parent cells with periodontitis-related stimuli, including mechanical stress and inflammatory factors, is an effective way to enhance the anti-inflammatory effects of CEVs, providing an easy-to-use and effective strategy for CEV modification.

### Regulation of cell proliferation, migration, osteogenesis, and angiogenesis

The proliferation, migration, and differentiation of endogenous stem/progenitor cells at an injury site, as well as angiogenesis, are indispensable steps in periodontal regeneration (Fig. 4). Chew et al. reported that MSC-derived CEVs promoted periodontal tissue regeneration by increasing cellular proliferation and migration. In vitro studies indicated that MSC-EVs activated prosurvival AKT and ERK signaling in PDL cells by presenting exosomal CD73 proteins [118]. In addition to MSC-EVs, CEVs derived from stem cells in apical papilla (SCAP-EVs) promoted the migration of HUVECs by transferring cell division cycle 42 (Cdc42) to these cells. As a result, SCAP-EVs accelerated palatal gingiva regeneration by promoting angiogenesis [119]. SHED-EVs have also been reported to promote the proliferation and migration of HUVECs and BMSCs and to enhance the angiogenesis-related differentiation of HUVECs and osteogenesis-related differentiation of BMSCs [120]. Additional mechanistic investigations suggested that the bioactivities of SHED-EVs were mainly due to the encapsulated Wnt3a and BMP2. By increasing nuclear β-catenin protein abundance and upregulating Smad1/5/8 phosphorylation, SHED-EVs activated Wnt/β-catenin and BMP/Smad signaling in stem cells respectively, and significantly promoted the osteogenic differentiation of BMSCs [120, 121]. In addition to engagement with osteogenic differentiation, miR-26a in SHED-EVs could promote angiogenesis via the TGF-β/SMAD2/3 pathway [122]. The combination of SHED-EVs with a β-TCP scaffold induced both neovascularization and new bone formation, thus promoting alveolar bone regeneration in a rat model of periodontal defects [120]. The biological effects of PDLSC-EVs (PDLSC-EVs) have been extensively investigated. In a rat model of periodontitis, PDLSC-EVs restored the osteogenic differentiation ability of PDLSCs in inflamed periodontal tissue by activating the Wnt signaling pathway, thereby accelerating bone healing [123]. By carrying miR-1246, CEVs induced angiogenesis by activating Smad 1/5/8 signaling [124].

The pro-osteogenic or pro-angiogenic effects of MSC-EVs can be enhanced via modification of their parent cells before CEV isolation. These modifications mainly include gene modifications, mechanical stimulation, and inflammation preconditioning. For example, Xu et al. recently showed that CEVs secreted by P2X7 receptor-encoding gene-modified PDLSCs rescued inflammation-impaired osteogenesis of PDLSCs. Gene modification could increase the abundance of miR-6747-5p, miR-6515-5p, and miR-3679-5p in CEVs, which could bind to the GREB-1 protein in different ways [125]. Lv et al. found that CEVs derived from osteocytes exposed to mechanical strain induced significantly increased proliferation of PDLSCs through the activation of miR-185b-5p/PTEN/AKT pathways and promoted osteogenic differentiation mediated via BMP/Runx2 in an inflammatory environment [126]. CEVs released from LPS-pretreated dental folic cells (DFCs) facilitated the migration, proliferation, and osteogenic differentiation of inflamed PDLSCs, accelerating periodontal regeneration in an inflammatory microenvironment [127]. Our group indicated that CEVs secreted by dental pulp stem cells compromised by periodontitis significantly enhanced angiogenesis via miR-378a they carried. Angiogenesis-related microRNAs (miRNAs) carried by CEVs could an silence Sufu expression in endothelial cells and activate Hedgehog/Gli1 signaling to stimulate endothelial cell proliferation, migration, and tube formation [128, 129].

Collectively, these studies highlight the multiplicativity of CEVs in regulating the behaviors of stem cells. Moreover, the bioactive effects of CEVs, such as pro-osteogenic and pro-angiogenetic effects of CEVs, can be modified and enhanced by preconditioning parental cells. Given that tissue regeneration is a complex and cascading process requiring coordinated control of multiple factors (e.g., cell migration, differentiation, and angiogenesis), CEVs represent promising agents for regenerative medicine, including but not limited to periodontal regeneration.

## The prospects for BEV-based therapeutic applications in periodontal regeneration

Although the pathogenic roles of BEVs in periodontitis have been widely studied and recognized, the potential applications of periodontopathogen-derived BEVs are still in their infancy. Compared with stem cell-derived EVs, BEVs show unique advantages such as cost-effective production. Large-scale isolation and purification of CEVs require extensive labor and time costs, which has hindered their clinical translation. In contrast, BEVs can be easily produced in cost-effective ways via large-scale cultivation of bacteria [130]. Specifically, bioreactors and hypervesiculating mutant strains have been successfully developed to increase the production of BEVs [131, 132]. To achieve widespread clinical use in periodontitis, a nonlife-threatening disease with a high prevalence, the treatment must be both cost-effective and scientifically sound [6]. From this point of view, the low cost and large-scale production of BEVs make BEV-based therapies more promising for periodontitis treatment.

As a bacteria-driven disease, periodontitis can be treated by abrogating the survival of periodontopathogens and preventing the formation of plaque biofilms. Due to the impermeability of the host cell membrane to antibiotics, working concentrations of antibiotics fail to kill intracellular bacteria [133, 134]. Notably, the periodontopathogens *P. gingivalis* and *A. actinomycetemcomitans* were grown intracellularly in human buccal epithelial cells [135], and antibiotics alone did not eliminate them [136]. Due to their high permeability with respect to host cells, BEVs can kill intracellular pathogens more effectively than antibiotics [137]. Compared with soluble antibiotics, such as gentamicin, BEVs have more killing power by delivering autolysin and peptidoglycan hydrolases [138, 139]. In addition, BEVs can be employed for antibiotic drug delivery to enhance the uptake of antibiotics, leading to superior antibacterial efficiency of conventional antibiotics [137]. Therefore, we suggest that BEVs be employed for antibiotic drug delivery or as potential antibacterial substances for effectively killing intracellular periodontopathogens. Moreover, BEVs can prevent the formation of plaque biofilms by inhibiting bacterial adhesion to host cells, further alleviating antibiotic resistance development [140]. Specifically, adhesins in BEVs inherited from their parent bacteria can inhibit bacterial adhesion to host cells. For instance, BEV-coated nanoparticles derived from *Helicobacter pylori* effectively inhibited *H. pylori* adhesion to gastric epithelial cell tissues in a dose-dependent fashion [141].

With bioengineering or detoxification, OMVs can also be used as biological carriers for vaccines. It has been reported that P. gingivalis OMVs retain the immunodominant determinant of P. gingivalis, as demonstrated by the increased production of salivary IgA, serum IgG and IgA in mice following intranasal administration [142]. The mucosal immune response elicited by P. gingivalis OMVs could further enhance the clearance of P. gingivalis in an oral infection model [143]. The strong immunogenicity of P. gingivalis OMVs is mainly derived from LPS and A-LPS-modified proteins in OMVs, and absorption of serum with LPS results in a dramatic reduction in immmunoreactivity [144]. Despite these progresses, the application of OMVs as vaccination is still in its infancy and extensive work is required to decrease their side effects.

## Challenges and future directions

The lack of standard and cost-effective methods for separating and purifying EVs represents a major barrier to EV-based therapies. Methods currently used to separate and purify EVs include ultracentrifugation, precipitation, ultrafiltration, chromatography, and immunoaffinity capture. Among these methods, separating EVs by ultracentrifugation is the gold standard method. However, from a clinical-scale manufacturing perspective, ultracentrifugation is not ideal because of its low productivity and purity outputs [24]. Tangential-flow filtration (TFF), a method for concentrating EVs from a medium based on size, is a promising method. Compared with ultracentrifugation, TFF concentrates up to 100-fold more EVs while enhancing the removal of unwanted albumin [145, 146]. Among the reported methods for EV purification, immunoaffinity capture might be the most promising method since it is based on antibody-coupled magnetic beads and can yield highly purified EVs [147]. Due to the heterogeneity of EVs, the surface protein composition of EVs with various origins can be quite different [148–150]. Among the reported surface markers, transmembrane proteins such as CD9, CD63, and CD81 have been widely utilized for immunoaffinity-based EV extraction [151]. Future EV manufacturing methods require both high yield and high purity, and a combined separation/purification approach may be better than single-step procedures. Therefore, among these methods, combinations of TFF and immunoaffinity capture or other steps for high purification represent the best methods to produce clinical-grade EVs.

The rapid clearance of EVs following systemic administration is also a challenge for EV-based regenerative strategies and cannot be ignored. After systemic administration, EVs can be cleared rapidly in the body, and most EVs cannot reach target sites to exert their bioactive effects [152]. Prolonging the retention of EVs in a desired area by immobilizing EVs within biomaterials may enhance EV therapeutic efficacy. Specifically, anchoring EVs in biomaterials can extend their bioactivity following administration, control their release, and potentially improve therapeutic efficacy [153–155]. More importantly, given the complexity of periodontal regeneration, which includes the oriented insertion of newly formed periodontal ligaments in bone and cementum, EVs alone cannot coordinate the signaling regulation needed during the aligned formation of the bone–ligament–cementum complex. However, well-designed multiphasic scaffolds with precise compartments for tissue regeneration and integration are promising for mediating spatiotemporal events during periodontal regeneration [156, 157]. Therefore, we believe that advanced multiphasic biomaterial scaffolds combined with spatiotemporally released bioactive EVs represent a potential application for periodontal regeneration.

In most studies, the pathogenic roles of EVs in periodontitis are recognized based on in vitro cell culture systems. Therefore, in vivo experiments are urgently required to provide strong evidence of their effects. Moreover, most studies use bulk separation of heterogeneous EVs obtained from culture medium or clinical samples to analyze the biological effects of EVs; however, effects due to the heterogeneity of the biology, structure, and function of single vesicles are generally not considered in these studies. Recent technological advances with in vivo imaging and single-vesicle analysis have enabled researchers to overcome these two main limitations and provide us with novel tools to study the roles of EVs in periodontal homeostasis at the single-vesicle level [148, 158]. Advancing our knowledge regarding the role of EVs in periodontal homeostasis based on these advanced approaches can shed new light on treatment options for periodontal disease.

## Conclusion and perspective

**Fig. 5:**
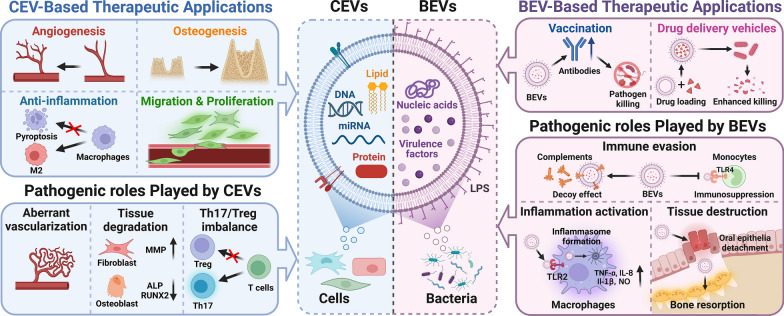
The roles of cell-derived extracellular vesicles (CEVs) and bacteria-derived extracellular vesicles (BEVs) in periodontal homeostasis. BEVs released from periodontopathogens contain virulence factors resulting in immune evasion, inflammation activation and tissue destruction. BEVs can also be used as biological carrier for vaccines and antibiotic drugs. CEVs released from cells contain bioactive cargos which can exert multiple biological effects, such as anti-inflammation, pro-migration & proliferation, pro-angiogenesis, and pro-osteogenesis. However, CEVs could also exert pathogenic effects under certain conditions. Created with BioRender.com

Overall, since EVs secreted by bacteria or cells play key roles in the interaction between the bacteria and host, the role of EVs in maintaining periodontal homeostasis is unquestionable. In this article, we thoroughly reviewed the pathogenic and therapeutic roles of BEVs and CEVs in periodontal homeostasis. By delivering different virulence factors, BEVs released from periodontal pathogens are envisaged as important missing pieces needed to reveal the imbalance in periodontal homeostasis and factors in periodontal disease progression. The multifunctional bioactivities of CEVs provide novel avenues by which to regain the loss of periodontal homeostasis and promote periodontal regeneration (Fig. 5). Despite great progress, the clinical translation of EVs is still in its infancy due to longstanding challenges, such as a lack of cost-effective production and rapid clearance in vivo. With the development of advanced biomaterials, the combination of EVs and multiphasic scaffolds holds great promise for periodontal regeneration.

## Data Availability

Not applicable.

## Abbreviations

EVs: Extracellular vesicles
BEVs: Bacteria-derived EVs
CEVs: Cell-derived EVs
PDLs: Periodontal ligaments
TLRs: Toll-like receptors
IL: Interleukin
RANKL: Receptor Activator for Nuclear Factor-κB Ligand
MVEs: Multivesicular endosomes
ILVs: Intraluminal vesicles
OMVs: Outer membrane vesicles
LPSs: Lipopolysaccharides
OIMVs: Outer-inner membrane vesicles
CMVs: Cytoplasmic membrane vesicles
PDLSCs: Periodontal ligament stem cells
Treg: Regulatory T-cell
PDLFs: Periodontal ligament fibroblasts
TNF-α: Tumor necrosis factor-α
HUVECs: Human umbilical vein endothelial cells
HGFs: Human gingival fibroblasts
ECM: Extracellular matrix
MCP-1: Monocyte chemoattractant protein-1
PRPs: Pathogen recognition receptors
CXCL: Chemokine (C-X-C motif) ligand
NOD1: Nucleotide-binding oligomerization domain-containing protein 1
CCL: Chemokine (C–C motif) ligand
MIP-1α: Macrophage inflammatory protein-1α
CDT: Cytolethal distending toxin
BMSCs: Bone marrow stromal cells
SHED: Stem cells from human exfoliated deciduous teeth
DPSC: Dental pulp stem cells
GMSCs: Gingival tissue-derived MSCs
SCAP: Stem cells in apical papilla
Cdc42: Cell division cycle 42
DFCs: Dental folic cells
TFF: Tangential-flow filtration
